# Medical students’ engagement in the context of the SARS-CoV-2 pandemic: The influence of psychological factors on readiness to volunteer

**DOI:** 10.3205/zma001506

**Published:** 2021-09-15

**Authors:** Luisa Mühlbauer, Johanna Huber, Martin R. Fischer, Pascal O. Berberat, Martin Gartmeier

**Affiliations:** 1 Technische Universität München, Fakultät für Medizin, Klinikum rechts der Isar, TUM Medical Education Center, München, Germany; 2 LMU München, Institut für Didaktik und Ausbildungsforschung in der Medizin (DAM) am LMU Klinikum, München, Germany

**Keywords:** medical students, SARS-CoV-2, volunteering in hospital, motivation, depression

## Abstract

**Objective: **To avert staff shortages during the first wave of the SARS-CoV-2 pandemic in spring 2020, the medical faculties of the Technical University of Munich (TUM) and the Ludwig Maximilian University of Munich (LMU) appealed to their students to volunteer for relief work. In this study, we examine the influence of psychological factors on the students’ decisions to respond to this call or not.

**Methodology: **We report on a cross-sectional study based on an online survey among medical students at the TUM and LMU. The survey consisted of a questionnaire containing items on motivation and other factors related to the decision for or against volunteering. Questions were also asked about anxieties regarding COVID-19 and the occurrence of depressive symptoms, as well as about resilience.

**Results: **Responses from 244 participants were analysed. Students’ decisions to volunteer revealed both altruistic and introjected motivations. For those students who did not volunteer, time overlaps and workload related to other activities played an important role. Between the two groups, no significant difference was detected in terms of their resilience and COVID-19-related anxieties. However, the non-volunteering students reported a significantly higher prevalence of depressive symptoms.

**Conclusion: **Sense of duty and the desire to help were, according to the students, the most important reasons for volunteering. Depressive symptoms and lack of time made volunteering less likely. Resilience and COVID-19-related anxieties do not seem to have had any influence on the decision to volunteer or not.

## Zusammenfassung

**Zielsetzung:** Um Personalengpässen im Zusammenhang mit der erste Welle der SARS-CoV-2-Pandemie im Frühjahr 2020 vorzubeugen, forderten die medizinischen Fakultäten der TU und der LMU München ihre Studierenden dazu auf, sich für einen freiwilligen Hilfseinsatz zu melden. Mit der vorliegenden Studie beleuchten wir den Einfluss psychologischer Faktoren hinsichtlich der Entscheidung der Studierenden, diesem Aufruf zu folgen oder dies nicht zu tun.

**Methodik: **Wir berichten eine Querschnittsstudie basierend auf einer Online-Umfrage unter Medizinstudierenden der TUM und der LMU München. Der Fragebogen enthielt Items zur Motivation sowie zu weiteren Rahmenbedingungen, die bzgl. der Entscheidung für oder gegen einen freiwilligen Hilfseinsatz relevant waren. Zudem wurden Fragen zu Ängsten bezüglich COVID-19, zum Auftreten depressiver Symptome sowie zur Resilienz gestellt.

**Ergebnisse: **Wir konnten die Antworten von 244 Teilnehmenden auswerten. Wichtige motivationale Faktoren bei den Studierenden für ein Meldung waren altruistische sowie introjizierte motivationale Motive. Bei der Gruppe der Studierenden, die sich nicht für einen Einsatz gemeldet hatten, spielten vor allem zeitliche Überschneidungen und die Auslastung durch andere Aktivitäten eine wichtige Rolle. Zwischen diesen beiden Gruppen konnte kein signifikanter Unterschied bezüglich ihrer Resilienz und ihren COVID-19 bezogenen Ängsten nachgewiesen werden. Die Gruppe der Nicht-MelderInnen berichtete jedoch eine signifikant höhere Prävalenz depressiver Symptome.

**Schlussfolgerung: **Pflichtgefühl und der Wunsch zu helfen waren den Angaben der Studierenden zufolge die wichtigsten Gründe, aus denen sie sich für einen Einsatz gemeldet hatten. Depressive Symptome und mangelnde Zeit haben eine Meldung unwahrscheinlicher gemacht. Resilienz und COVID-19 bezogenen Ängste scheinen keinen Einfluss auf die Entscheidung für oder gegen eine Meldung gehabt zu haben.

## 1. Introduction

At the beginning of 2020, the SARS-CoV-2 pandemic posed serious challenges to health systems worldwide. German health care institutions and decision-makers also had to meet these challenges as the numbers of cases rose sharply in March [1]. On 13 March 2020, the Federal Ministry of Health called on German hospitals to create capacity for COVID-19 cases [2]. A few days later, the medical faculties of the Ludwig-Maximilians-University (LMU) and the Technical University of Munich (TUM) contacted their medical students by email and appealed them to volunteer to support the health care facilities in coping with the crisis situation. The willingness of medical students to respond to this call helped to ensure that the first wave of SARS-CoV-2 infections in Germany was relatively well managed.

It was not actually certain that many students would answer this call: As for the rest of the population, the pandemic situation was initially unfamiliar and potentially threatening for them. It was impossible to predict how the situation in Germany would unfold. Dramatic television images, from Italy for instance, triggered anxiety in many people. In addition, the number of infections among health care workers was in places disproportionately high [3], [4], [5]. The medical students could not therefore foresee what volunteering for clinical relief work would entail. Against this background, we pursue two relevant questions in the present study: Firstly: What motivational factors were influential with regard to volunteering? Secondly: What influence did other psychological factors, specifically depression, anxiety and resilience, have in this regard? 

Finding answers to these questions is of interest and importance for several reasons: for one thing, it may help us react more effectively in the event of further waves of the current pandemic. The Robert Koch Institute’s situation report of October 2020 states: “Currently, there is an accelerated increase of transmission in the population in Germany. We therefore urgently appeal to the entire population to commit to infection control” [6]. According to the Academy of Medical Sciences, it is to be expected that the number of cases increases sharply again and that the health system could be confronted with additional problems, such as an accumulation of less acute cases [7]. Furthermore, epidemics or even pandemics will occur again in the future; society should be better prepared. In what follows, we explore the two key aspects of the study and their theoretical starting points. 

### 1.1. Motivational factors

Our inquiry into medical students’ motivation for volunteering in the first wave of the SARS-CoV-2 pandemic builds on existing research [8], [9], [10], [11], [12]. Such research explores the question of what supports and motivates volunteers in crisis situations that may stress them and even put them at risk. Current studies examine motivationally relevant factors in the context of voluntary services or voluntary crisis operations (e.g. the Ebola epidemic 2014-2016). One key finding is that a sense of duty and moral responsibility [11] are important motivational factors. Another study [13] is based on Clary et al.’s construct [14], which specifies six functions of volunteering (values, understanding, social, career, protective and enhancement functions). Medical students were asked about their motivation for volunteering. The values function (representing values involving an altruistic or humanitarian purpose) was found to be the most important for the students. A further study [15], in which students were also interviewed about the SARS-CoV-2 pandemic, highlighted a sense of duty and altruism as the most important factors influencing medical students’ motivation to volunteer in this situation. 

To conceptualise motivationally relevant factors, we refer to self-determination theory of motivation as formulated by Deci and Ryan [16], [17]. Their model distinguishes between external “extrinsic” and internal “intrinsic” motivation. Intrinsic motivation involves intrapersonal driving factors, such as pleasure in an activity or curiosity. Extrinsic motivation, on the other hand, derives from extrapersonal factors, such as rewards or punishments. In the most pronounced form of extrinsic motivation, the underlying drivers are completely external to the person; actions are carried out independently of or even in contradiction of internal attitudes. Between the two poles of external and intrinsic motivation, self-determination theory distinguishes the intermediate stages of introjection and identification. Introjected motivation is closer to external motivation; it may be regarded as an internalised form of external motivation, e.g. when the avoidance of guilty conscience is a motive for action. Identified motivation, on the other hand, is characterised by higher levels of intrinsic motivation. It involves greater conformity with a person’s inner attitudes. It is based on a deeper identification with an action or a goal [16], [17]. To capture these factors, we draw on preliminary work by Prenzel et al. [18] (see methodology section). The authors argue that the degree of individual interest in a particular area of content is also an important motivational factor. 

In this type of interest, intrinsic motivations are less important than the importance of a topic itself as the source of motivation. Following Prenzel et al. [18], we thus inquire into students’ interest and pleasure in volunteering (interest and intrinsic motivation), their professional ambitions as well as their sense of obligation (identified and introjected motivation). At the other end of the spectrum, we also examine external rewards (extrinsic motivation). 

In addition to these considerations, the study considered a complementary list of specific reasons for and against volunteering to address the particular circumstances of the SARS-CoV-2 pandemic. We assumed that factors such as lack of time or other personal circumstances would be plausible reasons for not volunteering.

#### 1.2. Depression, anxiety and resilience

Other possible psychological factors that may influence volunteering are considered below. Specifically, we look at the presence of depressive symptoms and COVID-19-related anxiety as well as resilience. 

According to [19], depression or a depressive episode is defined as the presence of two of three main symptoms: depressed mood, loss of interest and avolition over a period of at least two weeks, as well as the presence of at least two additional symptoms, such as reduced concentration or pessimistic future outlook. We hypothesise that existential anxiety and isolation may have been amplified in many of the respondents during the SARS-CoV-2 pandemic and may therefore have had a negative impact on mental well-being. Recent research on the prevalence of depressive symptoms during health crises [20], [21] shows, among other things, that depressive symptoms were more common among the population of Sierra Leone during the Ebola epidemic, and among affected people in Taiwan after the SARS epidemic. This leads us to ask whether there is a correlation between depressive symptoms and students’ unwillingness to volunteer. 

Furthermore, we investigate whether the circumstances of the SARS-CoV-2 pandemic triggered specific anxieties in the students. The uniqueness of the situation, particularly its rapid spread, media saturation, as well as the mandatory restrictions on daily activities [2], make this hypothesis plausible. In a study from Saudi Arabia conducted in connection with the occurrence of the MERS coronavirus, a significant proportion of the employees interviewed expressed concern about possible infection [22]. Furthermore, a correlation between risk-specific concerns related to an epidemic and increased protective action is assumed [23]. We therefore test the assumption that increased anxiety about COVID-19 could lead to more cautious and thus possibly hospital-avoidant behaviour. To this end, we use a questionnaire originally developed with regard to fears in the context of swine flu (H1N1 pandemic 2009) [24] and adapted to the current situation.

Finally, we examine the respondents’ resilience in terms of their psychological resistance to stressors. Resilience is understood as the ability to maintain agency and well-being despite stress [25]. The health professions in particular are subject to heightened burdens and stressors. Resilience as a protective factor is thus currently the subject of intensive research [26], [27]. We assume that the SARS-CoV-2 pandemic and the circumstances associated with it represented additional, salient stress factors with regard to the mental health of those affected [28]. Existing studies show that burnout and stress are very common among medical students [29], [30]. The question thus arises whether the students who showed a higher willingness to work in the hospital during the SARS-CoV-2 pandemic rated their resilience higher.

The present study investigates two questions: Firstly, what motivational factors influenced medical students to volunteer or not to volunteer at the hospital? Secondly, what role did fears about COVID-19, resilience and the presence of depressive symptoms in connection with the pandemic play with regard to volunteering or not volunteering?

## 2. Methodology

### 2.1. Ethical approval 

Participation in the study was voluntary and anonymous. Subjects were required to confirm an information sheet and informed consent form in order to participate. The ethics committee of the TUM reviewed and approved the study project (certification mark 281/20S).

#### 2.2. Participants and sample

The study target group was medical students enrolled in the clinical phase of their studies at the LMU and the TUM. A total of approx. 2,400 students in the clinical phase (approx. 900 at TUM and approx. 1,500 at LMU) were invited by email to participate in the survey.

#### 2.3. Study design and instruments

We conducted a multicentre cross-sectional study as an online survey. Students at the TUM and LMU were invited by email, on 14/05/2020 and 17/05/2020 respectively, to participate in the online survey. The survey was administered via the EvaSys platform and took place over a period of 36 days.

The questionnaire used largely validated and proven instruments. We used a motivation scale whose development was closely based on Prenzel et al.’s questionnaire [18], which examines various motivational factors in categories. The scale comprises 15 items on a six-point assessment scale (e.g. “The work is paid”). Additional questions were asked about the specific reasons for (seven items on a 4-point scale) or against (eight items on a 4-point scale) volunteering (see table 1 (Tab. 1) and table 2 (Tab. 2)). These items were also included in the questionnaire as a proprietary development. In this part of the questionnaire, respondents were given the opportunity to provide further reasons as free text. Furthermore, the German version of the 10-item Connor-Davidson Resilience Scale [25] was used to measure the participants’ resilience. This scale comprises ten items (e.g. “I am able to adapt when something changes”), which are assessed on a five-point scale. The Patient Health Questionnaire-9 (PHQ-9) [31] was used to assess depressive symptoms. It contains nine items (e.g. “Little interest or pleasure in your activities”), where agreement can be rated on a four-point scale. In addition, the Swine Flu Inventory [24] was adapted to assess participants’ anxiety about COVID-19. It was modified with regard to language (originally in English) and the SARS-CoV-2 pandemic and subsequently contained eight questions answerable on a five-point scale (see attachment 1 ). 

Using filter questions, the students were divided into two groups from the start of the questionnaire, depending on whether they indicated that they had volunteered in the context of the SARS-CoV-2 pandemic. The two groups of volunteers/non-volunteers were asked different questions. The volunteering group was asked questions about the factors that motivated them to volunteer. The non-volunteering group was asked what factors had prevented them from volunteering.

Both groups were given the 10-item Connor-Davidson Resilience Scale, the PHQ-9 and the adapted Swine Flu Inventory.

#### 2.4. Statistical analyses 

The statistical analyses were carried out using SPSS version 26. The students’ responses to motivational factors and demographic data were analysed descriptively in terms of absolute and relative frequencies, mean values and standard deviations. Cronbach’s alpha was calculated as a measure of internal consistency. Levene’s test was used to determine variance homogeneity in the mean comparisons. Group comparisons for the PHQ-9 and the adapted Swine Flu Inventory were conducted using unpaired t-tests. Due to lack of variance homogeneity, the 10-item Connor-Davidson Resilience Scale was evaluated using the Welch test. The significance threshold was set at 0.05.

## 3. Results

262 respondents participated in the study. 18 questionnaires were excluded due to lack of consent to the subject information and consent forms, no affiliation to the clinical study section or clearly irrational response patterns. Thus, 244 questionnaires (10% of the 2,400 students contacted) were available for evaluation. 94% of the usable responses were received within the first week. The demographic data of the sample are summarised in table 3 (Tab. 3). The groups showed no marked differences with regard to the characteristics shown here. Female medical students are slightly overrepresented in the survey; the average proportion of female medical students is slightly lower at approximately 62% [32].

The verification of the scales used showed high internal consistency of the items on depressive symptoms (Cronbach’s alpha=.80). The resilience scale was found to be reliable, with a Cronbach’s alpha=.82. Only moderate internal consistency was found for the eight items on anxiety related to COVID-19, with a Cronbach’s alpha=.56. This limitation is considered in the interpretation and discussion of the results below.

### 3.1. Motivation of volunteers

Of the volunteering group, 120 of the 197 students (61%) were eventually mobilised. The activities performed by the students surveyed were either medical (25%), nursing (39%) or organisational (36%) in nature. The students’ places of assignment were varied and included normal wards, intensive care units, corona screening wards, emergency rooms, public health departments, and laboratories. Regarding the frequency of contact with COVID-19 patients during their volunteering, students reported the following: 29%=no contact; 42%=occasional contact; 29%=daily contact.

**Figure 1:**
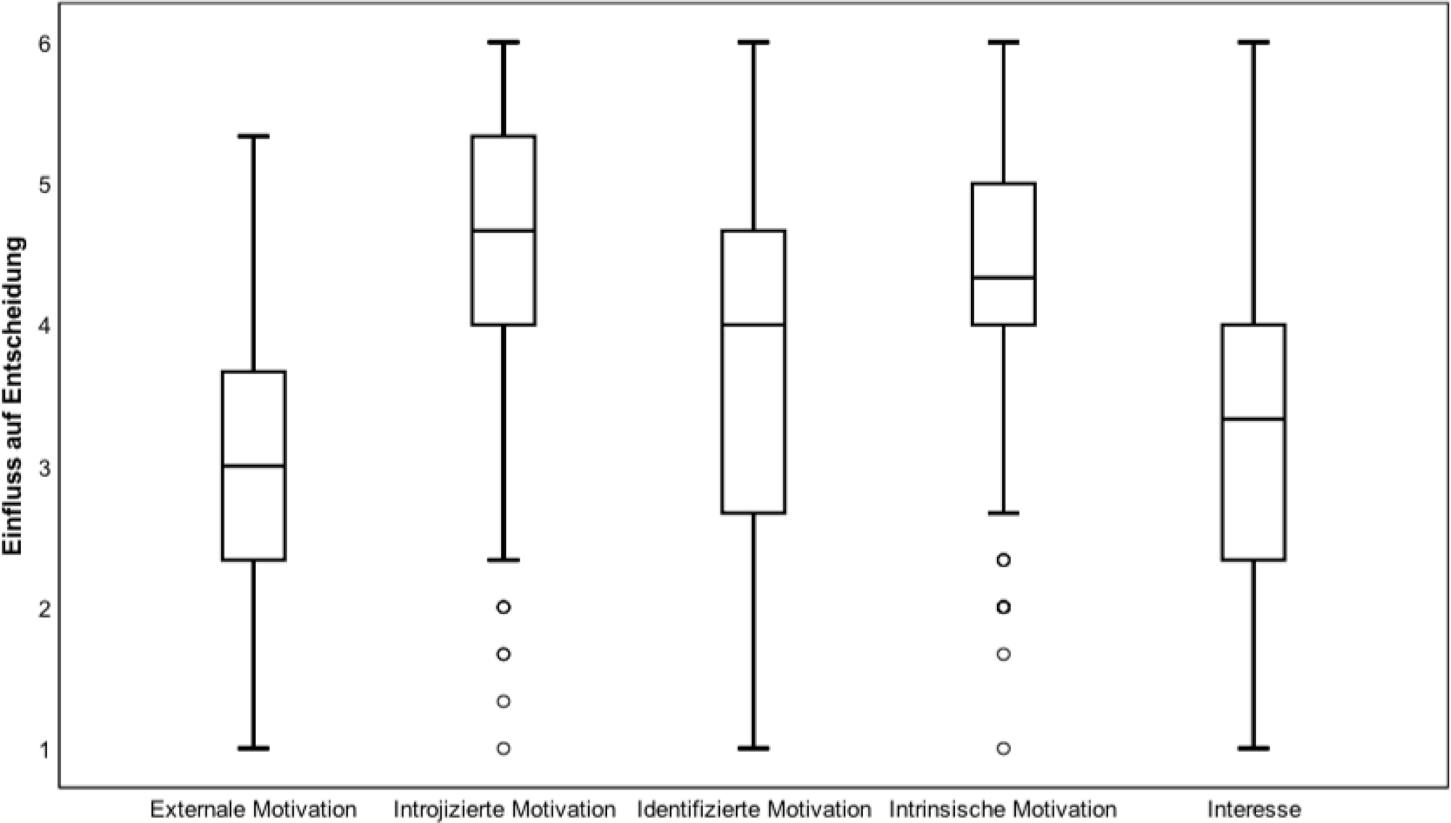
Comparison of the influence of the different types of motivation on the decision to volunteer in the corona crisis

Figure 1 (Fig. 1) shows the influence of the different types of motivation on the decision to volunteer during the Corona Crisis. Introjected motivation was the most influential factor for this group (median=4.7, M=4.5, SD=1.1). This includes motivational factors such as “It’s part and parcel of being a medical student.” Respondents assessed their intrinsic motivation along the same lines (median=4.3, M=4.4, SD=1.0). Items included “I am happy to be able to help in the current situation.” The mean value was somewhat lower in the area of identified motivation (median=4.0, M=3.7, SD=1.3, example item “It will be a step towards reaching my professional goals.” The facets that had the least influence on the students’ decision were interest (median=3.3, M=3.2, SD=1.2, sample question “I wanted to get involved in stimulating tasks that I have always wanted to learn more about”) and external motives (median=3.0, M=3.0, SD=1.0, sample question “The work will be paid”). 

#### 3.2. Reasons for or against volunteering

Table 1 (Tab. 1) and table 2 (Tab. 2) show the specific reasons against or for volunteering during the SARS-CoV-2 pandemic. A comparison of the tables makes clear which additional factors played a role in the decision-making process. For example, the reason “Other professional commitments” was the most negative influence on the decision to volunteer. Another noteworthy factor is that “fear of increased risk of infecting friends or family” was rated as markedly more decisive than “fear of becoming infected myself”. In table 2 (Tab. 2), it is noticeable that “helping to combat a major social crisis” was rated as a very important reason for volunteering.

All the students surveyed were able to provide additional reasons in a free text field (16 respondents in the non-volunteering group, 24 in the volunteering group). The group of non-volunteers often listed very specific reasons for not being available. Commitments due to clinical traineeships or a doctoral thesis were mentioned here, but health and organisational obstacles were also cited. 

In the volunteering group, more than half of the answers consisted of generally formulated, altruistic motives, such as a feeling of social obligation. “I simply wanted to help and was glad that I was able to do so thanks to my studies”, or “All assistance was urgently needed, I am young and feel obliged to help my fellow human beings especially since I have the necessary knowledge to do so! The older the people, the more important it is to stay off duty. I wanted to relieve them of the responsibility” are typical responses. Other answers also mentioned the possibility of earning money and gaining credit towards their studies. 

#### 3.3. Depression, anxiety and resilience as further psychological influences

When focussing on the differences between the two groups with regard to the psychological factors presented in table 4 (Tab. 4), it can be seen that with regard to participants’ anxiety, there was no significant difference with regard to COVID-19 t(222)=1.7, p=.088. Comparing resilience also shows no significant difference t(240)=1.6, p=.223. It was only in the analysis of depression that the non-volunteering group displayed significantly higher sum values t(239)= -2.0, p=.042. Depressive symptoms were slightly more pronounced on average among those students who had not volunteered. 

## 4. Discussion

Our results indicate that in the first wave of pandemic it was primarily altruistic motives and the desire to help that motivated the medical students to volunteer. Among the volunteering group, introjected motivation was found to be the most prevalent type of motivation. For the non-volunteering group, time overlaps and commitments to other activities were the most important reasons for not volunteering. In respect of resilience and COVID-19-related anxiety, no significant difference was found between the two groups of students. It was only with regard to depression that the non-volunteering group displayed more symptoms.

### 4.1. Limitations

The pandemic situation developed dynamically during the survey timeframe. In particular, the many headlines on it could therefore have had an effect on students’ anxieties about COVID-19, depending on the day in question. In order to achieve the highest possible comparability within the students’ responses, we refrained from sending a reminder email or a second invitation to participate in the survey after the initial mail to participate. Since 94% of the responses arrived in the first week of the survey, we assume that the students answered our questions under largely similar conditions.

A possible limitation of the validity of the data for some questions is the influence of social desirability bias. The question “Volunteering looks good on my CV” for example, was rated as the least important factor in relation to students’ motivation. Another possible distortion of results could be a volunteer bias resulting in the overweighting of students volunteering at the hospital. In our sample, this was the larger group. It is likely that these students had a particularly strong interest in the topic. At the same time, it is conceivable that non-volunteers tended to abstain from the survey for fear of not having acted in a socially desirable manner. Furthermore, it is possible that “non-volunteering” and “non-participation” in the study were based on the same individual reasons (someone who does not report due to time constraints probably also considers their time budget too tight to participate in the study), which would indicate a correlation between the two behaviours. This may also be the reason for the significantly lower response rate (ergo sample size) of the non-volunteering group. However, since we do not derive any generalised statements about both groups, we can assume there is no substantial distortion of the group-specific results. 

#### 4.2. Interpretation

In the present study, introjected and intrinsic motivation were found to be very important for the students. Even though our study took place under unprecedented conditions, our findings are in line with those of Fletcher and Major [13], who investigated different functions of volunteering.

The study by Tempski et al. [15] and Kpanake et al. [11], in which the medical students’ sense of duty and altruism and sense of duty and moral responsibility were identified as the most important factors influencing motivation, also tend to coincide with the observations of our study. In addition to the salience of the motivational factors, this is also expressed in the free-text responses, in which both the desire to help and the sense of duty factors were frequently mentioned.

The external motivation of the volunteering group was found to be the lowest in our study. It therefore seems reasonable to assume that external incentives hardly played a role for the students in the context of the present situation, given that motivation tended to originate from the students themselves. However, the possible influence of social desirability bias on the results must also be considered here.

Feelings of belonging and autonomy may promote further internalisation of motives [17], ultimately strengthening introjected and identified motivation. In addition, students’ interest could be fostered by expanding teaching related to SARS-CoV-2 and increasing representation of general health crises in the curriculum. Stronger external motivation might be increased through external incentives, for example by crediting students’ voluntary work in the hospital as a course achievement. Greater recognition of volunteering could also have a positive effect on the motivation of students to help. Since the time factor was the main limiting factor for the non-volunteering group, higher remuneration could have a positive effect. This would make it possible to compensate for the loss of additional income from a part-time job. A similar effect could be achieved by reducing the study-related workload in favour of hospital work or by crediting hospital work as a study achievement. Such possibilities were created at the LMU and the TUM, where the students were able to declare their work via a self-disclosure form and have it credited as coursework. 

The statistically non-significant difference between the groups with regard to their COVID-19-related anxieties is contrary to our original assumption that anxiety could be one of the main factors preventing students from volunteering. However, the certainty of interpretation is weak here due to the low Cronbach’s alpha. The only evident tendency is that anxiety played some role with regard to COVID-19. Although anxiety in the population has increased due to the SARS-CoV-2 pandemic [28], medical students surveyed reported low levels of anxiety about SARS-CoV-2 infection [33]. On the other hand, our results show that the students’ fear of infecting people in their environment is significantly higher (cf. table 1 (Tab. 1)).

Furthermore, our study did not show any significant influence of resilience on the decision for or against volunteering. The students tended to rate their psychological resilience high across both groups. The recorded value of 29.4 was in a similar range to comparable study populations (27.8 in Montero-Marin et al. [34] and 30.1 in Hartley [35]). 

One interesting finding was the statistically significant difference between the two groups regarding the prevalence of depressive symptoms. Here, the non-volunteers reported substantially higher levels of depression. Since the anxieties related to COVID-19 tended to be similar between the groups, the specific threat does not seem to have been a decisive factor in the occurrence of depressive symptoms. Questionnaire studies conducted in the general population during the Corona lockdowns in spring 2020 indicate an increased prevalence of a number of mental disorders [28], [36]. That the volunteering group in the present study was less affected by such problems is plausible for several reasons. For example, the relationship between depression and (especially physical) activity is well established [37], [38]. Volunteering was naturally associated with activity for the students, which could have played a role in the prevention of depressive symptoms. Links to the self-determination theory of motivation [16], [17], [18] have already been made here; this approach also describes social involvement as an important source of individual motivation. Volunteering is associated with social contacts and social exchange and can therefore forestall depressive episodes. The prevention of depressive symptoms is an objective to which universities and faculties would do well to address. Preventive and supportive support services should be expanded on the part of faculties and universities. This could have a positive influence on the willingness of students to engage in voluntary work. 

Overall, the willingness of medical students to volunteer in spring 2020 was high. At the TUM, for example, more than half of all students contacted agreed to volunteer within eight weeks - significantly more than could be mobilised during the first wave of the pandemic. In the event that the willingness to volunteer in a future, comparable situation is lower or the need for supplementary relief is significantly greater, it is useful to know what motivated the students to volunteer first time around. Whether and in what form medical students are included in activities related to the SARS-CoV-2 pandemic varies between countries. In addition, a variety of factors and arguments shaping and influencing this decision are conceivable [39]. Hence, the attitude and motivation of medical students are also of interest in informing future policy and practice. In view of the general staff shortages in hospitals [40], it is therefore well worth studying what motivates students to volunteer in a crisis or what discourages them from doing so.

## 5. Conclusion

Whether or not medical students volunteered for hospital duty is related to their available time, sense of duty and desire to help, among other factors. Similarly, volunteering is more likely in the absence of depressive symptoms. In terms of resilience and anxiety regarding COVID-19, there seems to be no correlation with volunteering or not volunteering. 

## Competing interests

The authors declare that they have no competing interests. 

## Supplementary Material

Attachment 1

## Figures and Tables

**Table 1:**
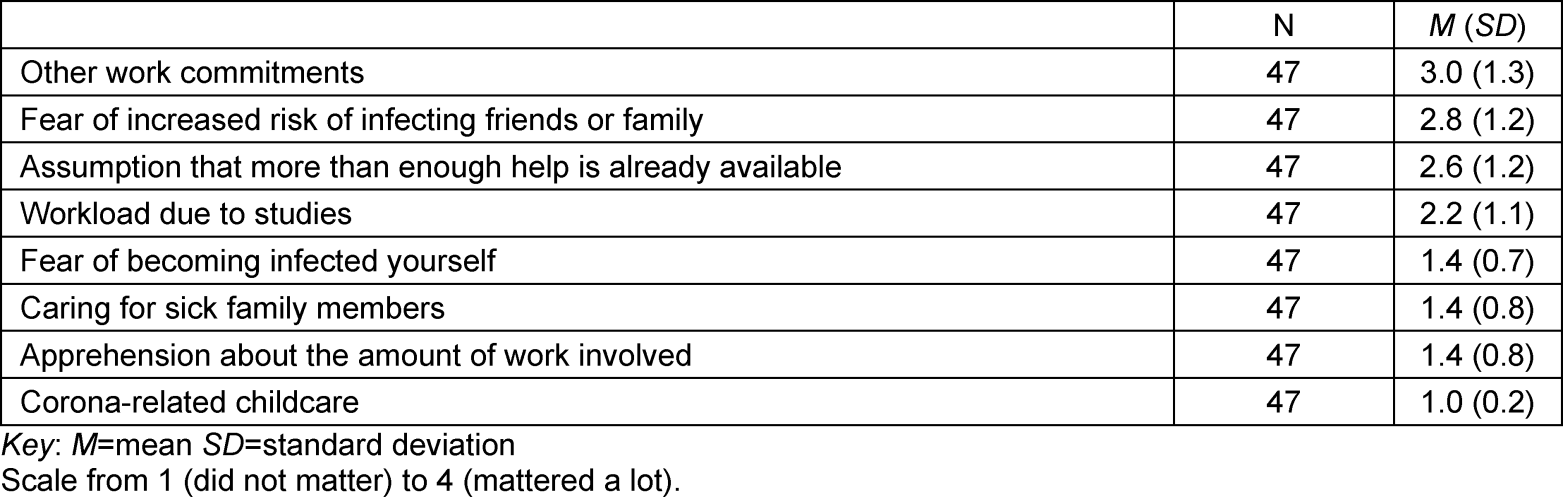
Reasons against volunteering during the SARS-CoV-2 pandemic

**Table 2:**
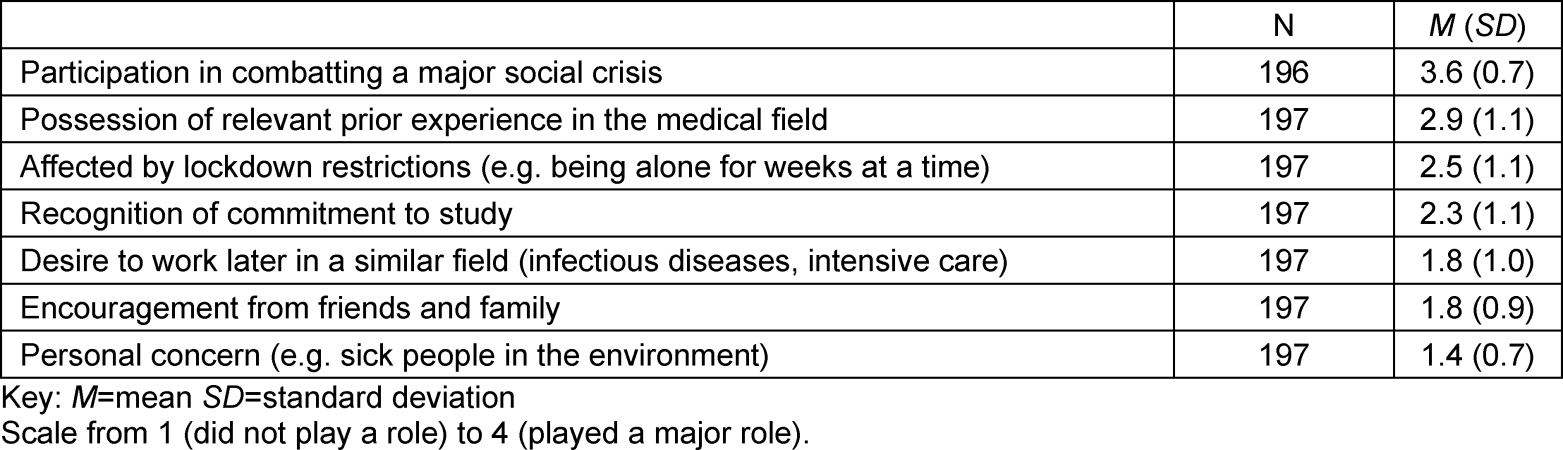
Reasons for volunteering during the SARS-CoV-2 pandemic

**Table 3:**
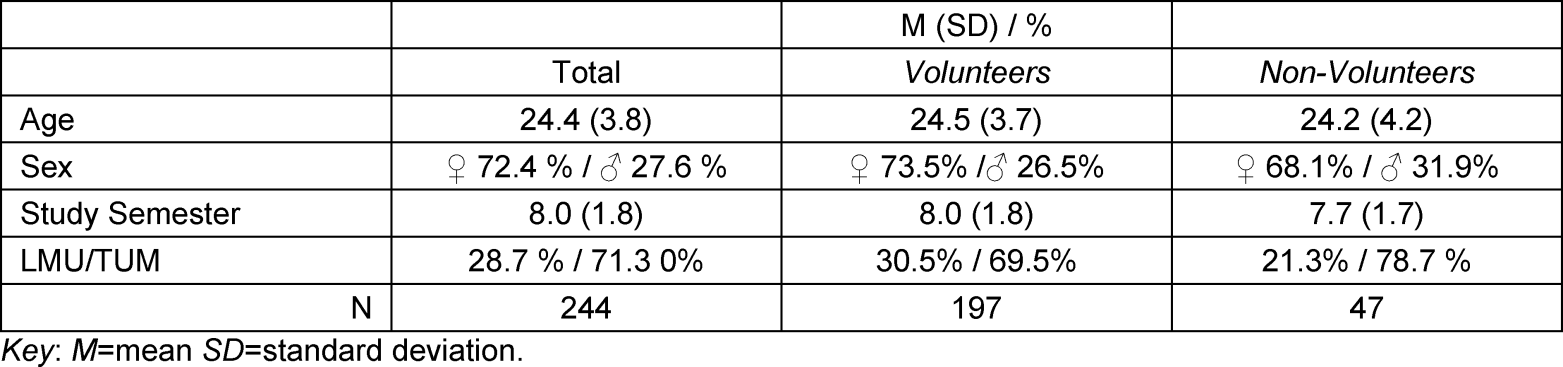
Demographic data for the students

**Table 4:**
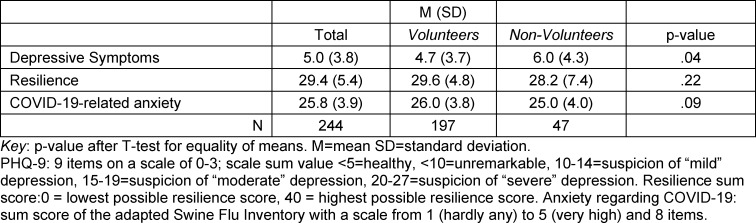
Comparison of the volunteering and non-volunteering groups for depressive symptoms, resilience and anxiety related to COVID-19
