# Supplementary material for: Medical students’ engagement in the context of the SARS-CoV-2 pandemic: The influence of psychological factors on readiness to volunteer
Source: GMS J Med Educ. 2021 Sep 15;38(6):Doc110. doi: 10.3205/zma001506 (PMC8493846; doi:10.3205/zma001506)
Supplement: Attachment 1 [file JME-38-6-110-s-001.pdf]

Attachment 1:

Items adapted from the Swine Flu Inventory [24]:

1. “To what extent are you concerned about COVID-19 disease?”
2. “How likely are you to become infected yourself?”
3. “How likely is it that someone you know will become infected?”
4. “If you have become infected, to what extent are you worried that you will become seriously ill?”
5. “To what extent has the risk of Corona infection influenced your decisions to be around other people?”
6. “To what extent has the risk of Corona infection affected your use of safety measures (e.g. disinfectants)?”
7. “How quickly do you think the infection will spread in the future?”
8. “How much information have you obtained regarding COVID-19 disease?”

Items to capture motivation based on [18]:

External motivation

- The work is paid
- I answered the call from the clinic management
- Volunteering looks good on my CV

Introjected motivation

- I would have had a guilty conscience if I did not volunteer
- It’s the right thing to do as a medical student
- I wanted to feel I was doing the right thing

Identified motivation

- I want to learn things that I need to be able to do for my job
- I want to experience the current situation in a clinic first hand
- It is a step towards achieving my professional goals

Intrinsic motivation

- I am happy to help in the current situation
- I am very interested in the current situation, so I want to be actively involved
- I was curious about how work is actually done in the clinic.

Interest

- I wanted to engage in stimulating tasks that I have always wanted to learn more about
- I wanted to satisfy my curiosity and to develop myself
- I wanted to see and learn interesting things that I can share with others

Attachment 1 to: Mühlbauer L, Huber J, Fischer MR, Berberat PO, Gartmeier M. *Medical students’ engagement in the context of the SARS-CoV-2 pandemic: The influence of psychological factors on readiness to volunteer.* GMS J Med Educ. 2021;38(6):Doc110. DOI: 10.3205/zma001506
